# Shaping the dynamic mitochondrial network

**DOI:** 10.1186/1741-7007-12-35

**Published:** 2014-05-27

**Authors:** Laura L Lackner

**Affiliations:** 1 Department of Molecular Biosciences, Northwestern University, 2205 Tech Drive Hogan 2-100, Evanston, IL 60208, USA

## Abstract

In a majority of cell types, mitochondria form highly dynamic, tubular networks. Maintaining the shape of this complex network is critical for both mitochondrial and cellular function and involves the activities of mitochondrial division, fusion, motility, and tethering. Recent studies have advanced our understanding of the molecular mechanisms underlying these conserved activities and their integration with cellular needs.

## 

Mitochondria are not discrete or autonomous but form highly dynamic, interconnected networks whose biogenesis and structure are highly influenced by the needs of the cell (Figure 1a,b). Mitochondria have a myriad of functions in addition to cellular energy production and play critical roles in cell cycle progression, differentiation, development, immune responses, lipid and calcium homeostasis, and apoptotic cell death (Figure 1c; reviewed in [1]). These diverse roles of mitochondria are intimately connected to the structure and cellular context of the essential organelle. Thus, it is not surprising that aberrant mitochondrial architecture has been associated with an ever-increasing number of diseases.

**Figure 1:**
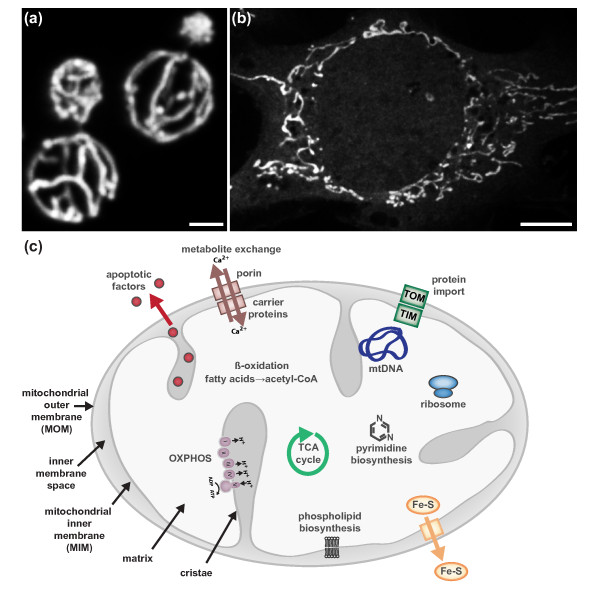
**The form and functions of mitochondria. (a)** In yeast, mitochondria form a connected, tubular network that is evenly distributed at the cell cortex. **(b)** Mitochondria also form well-distributed tubular networks in a majority of mammalian cell types. The mitochondrial network of a mouse embryonic fibroblast is shown. Scale bar, 2 μ for (a,b). **(c)** Like their bacterial ancestors, mitochondria possess two structurally and functionally distinct membranes, the mitochondrial outer and inner membranes (MOM and MIM, respectively). The MOM and MIM surround two compartments, the inner membrane space and matrix, respectively. The matrix houses the circular mitochondrial genome (mtDNA), which encodes protein components of the respiratory complexes I to IV. The MIM, the most protein dense membrane in the cell, adopts elaborate folds called cristae in which assembled respiratory complexes are housed. In addition to ATP production via oxidative phosphorylation, mitochondria play critical roles in phospholipid biosynthesis, metabolite exchange/buffering, β-oxidation of fatty acids, iron-sulfur cluster biogenesis, pyrimidine biosynthesis and the storage and release of apoptotic factors (reviewed in [1]). TCA, tricarboxylic acid.

The shape and cellular distribution of the mitochondrial network is maintained in large part by the conserved activities of mitochondrial division, fusion, motility and tethering (Figure 2). These conserved activities are coordinately regulated and fully integrated with cellular physiology to respond to the rapidly changing needs of the cell. For example, mitochondria elongate during the G1/S transition, fragment at the onset of mitosis and apoptosis, hyperfuse in response to nutrient starvation and oxidative stress, and are recruited to and maintained at active synapses [2-8]. This regulated restructuring of mitochondria is functionally significant as disruption of these processes has negative effects on overall cellular function.

**Figure 2:**
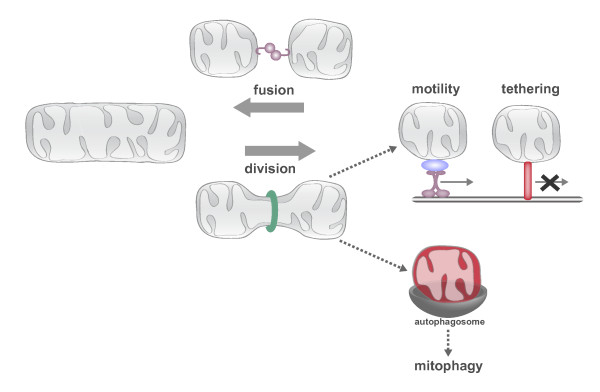
**The conserved activities of mitochondrial division, fusion, motility and tethering shape and position the dynamic mitochondrial network.** The connectivity of the mitochondrial network is controlled by the antagonistic activities of mitochondrial division and fusion. Mitochondrial division and fusion serve to create a compartment that is a connected conductor, able to mix its contents and have access to mtDNA and its products, but able to be distributed to distant cellular destinations via motor-dependent transport on actin or microtubule networks. Once transported to areas of demand, tethers ensure mitochondria are retained at these cellular locations. In addition to creating transportable mitochondrial compartments, mitochondrial division can produce functionally asymmetric daughter mitochondria. Dysfunctional daughters (depicted in red) cannot re-fuse with the network and are flagged for autophagic degradation.

Great progress has been made in our understanding of the molecular mechanisms that actively shape the dynamic mitochondrial network. However, we still have much to learn regarding the coordinate regulation of the activities that drive the context-specific changes in mitochondrial form and function. This review will highlight recent advances in our understanding of the molecular mechanisms that impact mitochondrial form and the integration of these mechanisms with one another and with cellular function.

### Mitochondrial division and fusion: regulators of mitochondrial connectivity

The antagonistic activities of mitochondrial division and fusion are required to maintain the form and function of mitochondria (Figure 2). Mitochondrial fusion facilitates communication and sharing of contents between mitochondrial compartments, which can buffer transient defects in mitochondrial function [9]. Mitochondrial division facilitates the transport, distribution, and quality control-mediated degradation of the organelle [10]. The dynamic processes of mitochondrial division and fusion are mediated by dynamin related proteins (DRPs). DRPs are a family of large GTPases that harness GTP-dependent self-assembly and subsequent GTP hydrolysis-mediated conformational changes to remodel membranes [11,12]. The DRP Dnm1/Drp1 (yeast/mammals) drives the scission of mitochondrial membranes, and the DRPs Fzo1/Mfn1/2 and Mgm1/Opa1 mediate fusion of the mitochondrial outer and inner membranes (MOM and MIM), respectively [13].

#### Mitochondrial division

The dynamin related GTPase Dnm1/Drp1 is a core component of the mitochondrial division machine (Figure 3a) [14-18]. Dnm1/Drp1 assembles into helical structures that wrap around mitochondria and mediate the scission of mitochondrial membranes [17,19-21]. GTP binding drives Dnm1/Drp1 helix assembly, which in turn triggers GTP hydrolysis via the formation of a catalytic interface between the GTPase domains of molecules in adjacent helical rungs [19,22-25]. Consequent GTP hydrolysis-driven conformational changes in the helix result in further constriction and ultimate scission of the underlying mitochondrial membranes [26,27].

**Figure 3:**
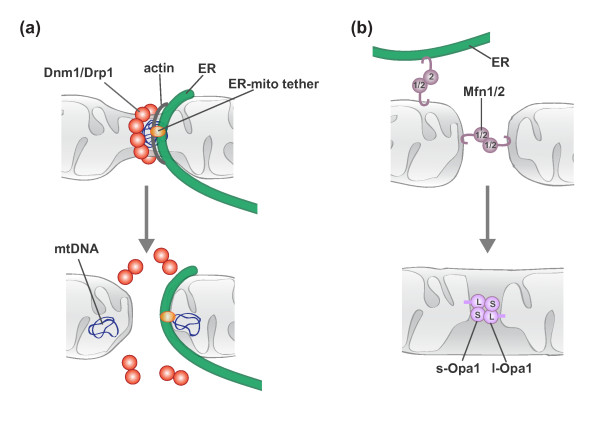
**Molecular models of mitochondrial division and fusion. (a)** Mitochondrial division involves communication between extra-mitochondrial division factors and internal mitochondrial structures. See text for details. **(b)** Mitochondrial fusion requires the sequential interaction of the MOM and MIM. MOM fusion is mediated by Mfn1/2, and MIM fusion is mediated by Opa1. Mfn2 is also localized to the ER and functions to tether the ER and mitochondria.

Drp1-mediated membrane remodeling is subject to regulation at various points in the division pathway, including targeting of Drp1 to the MOM and modulation of the GTP-regulated assembly, constriction, and disassembly of the Drp1 helix. Post-translational modification (PTM) of Drp1, which includes phosphorylation, sumoylation, ubiquitination, nitrosylation and O-glycosylation, can positively or negatively impact Drp1 activity (reviewed in [28]), and alternative RNA splicing produces functionally distinct Drp1 isoforms, which themselves are subject to differential PTM [29]. The activity of the mitochondrial division DRP is also regulated by protein effectors (adaptors): Mdv1 in yeast and Fis1, Mff, MiD49 and MiD51 in mammals [30-36]. These effectors function to target and/or regulate the assembly of the division DRP on the mitochondrial surface, providing critical spatial and temporal regulation [37-40].

Distinct combinations of Drp1 isoforms, effectors and PTMs provide contextual regulation to Drp1 activity and allow for the integration of mitochondrial division with cellular needs. For example, site-specific phosphorylation activates Drp1 activity during mitosis, which facilitates segregation of mitochondria into daughter cells [3]. During nutrient starvation, the phosphorylation of one site and dephosphorylation of another attenuate Drp1 activity, leading to mitochondrial elongation, which protects mitochondria from autophagic degradation and sustains cell viability [4,5]. Phosphorylation has recently been shown to regulate the association of splice-specific isoforms of Drp1 with microtubules (MTs) [29]. MT-associated Drp1 is a latent form of Drp1 that can be selectively mobilized by cyclin-dependent kinase signaling, providing a novel mechanism to integrate Drp1 activity with the cell cycle. In addition, recent structural analyses of the Drp1 effector MiD51 identified ADP as an unexpected regulator of mitochondrial division [38,41]. MiD51 adopts a nucleotidyltransferase fold that can bind ADP. MiD51 mutants deficient in ADP binding are able to recruit Drp1 to mitochondria [38,41] and support mitochondrial division under normal conditions [41]. However, stress-induced mitochondrial division is attenuated in the absence of ADP-binding [38], providing context-specific regulation to the activity of the division effector.

While protein and PTM effectors can function to target the mitochondrial division DRP to the mitochondrial surface, the amalgamation of recent studies indicates that mitochondrial division site selection involves communication between unexpected extramitochondrial factors and internal mitochondrial structures. The initial observation that the ER physically wraps around mitochondria and facilitates mitochondrial constriction at nascent division sites added a novel player to the mitochondrial division pathway [42]. ER-mediated mitochondrial constriction occurs upstream of Drp1 recruitment and represents an early stage in mitochondrial division [21,42]. In mammalian cells, an ER-associated formin, INF2, has been functionally linked to this early stage mitochondrial constriction [43]. Evidence indicates that INF2 mediates actin polymerization and subsequent myosin recruitment to sites of ER-mitochondria contact, providing a force-generating mechanism to drive the constriction of mitochondria [44]. This initial constriction likely generates a geometric hotspot that is more favorable for Drp1 helix assembly [42]. Evidence hints that actin may play a similar role in mitochondrial division in yeast. In yeast, the multi-subunit ER-mitochondria encounter structure (ERMES), which tethers the ER and mitochondria, is both spatially and functionally linked to sites of mitochondrial division [45]. The ERMES complex has also been implicated as a bridge between mitochondria and the actinomyosin network and thus may function at division sites to coordinate the recruitment of cytoskeletal and motor proteins [46].

The spatial and functional link between ERMES and mitochondrial division also place a matrix-localized factor, the mitochondrial nucleoid, at the site of mitochondrial division. The mitochondrial nucleoid is composed of mitochondrial DNA (mtDNA) and proteins required for its compaction and maintenance. A subset of mitochondrial nucleoids, specifically those that are actively replicating, co-localize with the ERMES complex [47]. ERMES-associated nucleoids are present at a majority of mitochondrial division sites, and nucleoids are often found in both of the newly generated mitochondrial tips following division [45]. As tips can go on to fuse with other parts of the mitochondrial network, the placement of nucleoids in newly generated mitochondrial tips provides a means to redistribute mtDNA within the network. The spatial association of nucleoids with mitochondrial division sites is conserved, and defects in mitochondrial division lead to defects in nucleoid distribution in both yeast and mammalian cells [48,49]. While homologs of the core ERMES subunits are not found in higher eukaryotes, the putative ERMES regulatory subunit Gem1 (Miro 1 and 2 in humans) is conserved [50,51]. Whether Miro associates with and regulates a functionally analogous ER-mitochondria tether in higher eukaryotes is an outstanding question. It is likely additional factors that mediate physical and functional interactions between matrix-localized mitochondrial nucleoids and the MOM-associated division machinery will be identified. Indeed, based on the recent addition of novel, unexpected players to the mitochondrial division pathway in both yeast and humans, it is clear that our knowledge of the entire complement of proteins that comprise and regulate the division complex is far from complete.

#### Mitochondrial fusion

In comparison to the division DRPs, less is known about the mechanism by which fusion DRPs harness GTP-driven self-assembly and GTP hydrolysis-mediated conformational changes to fuse membranes. Current evidence indicates that MOM and MIM fusion proceed via two separable stages, membrane tethering and lipid content mixing, both of which require fusion DRPs (Figure 3b) [52-58]. Membrane tethering is mediated by fusion DRP self-assembly, and subsequent GTP hydrolysis-induced conformational changes are proposed to destabilize the lipid bilayers of the tethered fusion partners to facilitate lipid mixing and fusion. While assembled structures of the fusion DRPs have been observed by electron microscopy, it is not yet clear if and how these structures correlate to active fusion complexes [59-62].

Like the mitochondrial division DRP, the mitochondrial fusion DRPs are subject to various levels of regulation, including alternative splicing, PTM, proteolytic processing, and regulated protein degradation, all of which can link mitochondrial fusion with cellular physiology. For example, one of the peptidases responsible for Opa1 processing, Oma1, has been shown to be activated in response to various cellular stressors [63]. Under these conditions, enhanced Opa1 processing correlates with attenuated fusion and stress-induced mitochondrial fragmentation. In contrast, OXPHOS-stimulated processing of Opa1 via the peptidase Yme1L has been shown to stimulate MIM fusion [64]. Recent work demonstrates that acetylation can also regulate Opa1 activity. Acetylation of OPA1 reduces its activity, and the acetylated state and thus function of OPA1 can be modulated by the mitochondrial deacetylase Sirt3 [65]. As the sirtuin is dependent on NAD^+^, its activity is highly sensitive to the metabolic state of the cell. Thus, context-specific processing and PTM of Opa1 link MIM fusion to both cellular health and metabolism. Acetylation is also proposed to regulate MOM fusion under certain stress conditions by promoting the ubiquitination and subsequent degradation of Mfn1 [66]. Phosphorylation and ubiquitination can also trigger Mfn1/2 degradation and thus inhibit MOM fusion in response to specific stimuli [67-69]. In addition to promoting degradation, site-specific ubiquitination can stabilize MOM fusion DRPs, as has been shown for Fzo1, and promote fusion, perhaps via the stabilization of Fzo1 oligomers [70]. Regulation of the oligomeric state of Mfn2 via modification by oxidized glutathione has also been proposed to promote mitochondrial fusion [71]. Additionally, localization of Mfn2 to both the MOM and ER raises the possibility that differential targeting of the protein can also be used as a means to regulate fusion. As interactions between ER-associated Mfn2 and mitochondrial-associated Mfn1/2 function to tether the two organelles [72], ER-mitochondria contacts may also play direct and/or regulatory roles in mitochondrial fusion. Given the complex regulation of mitochondrial fusion at the level of both the MOM and MIM, and the likelihood that additional regulatory mechanisms will be identified, we have a challenging road ahead in the pursuit of a complete understanding of how mitochondrial fusion is fully integrated with cellular needs.

#### The integration of mitochondrial division and fusion

The relative rates of mitochondrial division and fusion modulate the connectivity of the mitochondrial network. Under normal conditions, the rates of mitochondrial division and fusion in yeast are balanced, suggesting that there is coordinate regulation of the two processes [73]. A reported interaction between Drp1 and Mfn2 raises the possibility that mitochondrial dynamics may be coordinated via direct interactions between the division and fusion machinery [74]. Further support of coordinate regulation comes from recent work that both spatially and functionally links the short form of Opa1 to mitochondrial division [75]. Non-processed forms of Opa1 (long Opa1) are sufficient to mediate mitochondrial fusion, while the accumulation of processed forms (short Opa1) correlate with mitochondrial fragmentation without negatively effecting fusion rates, indicative of a role for short Opa1 in division. Consistently, a catalytically inactive form of short Opa1 partially co-localizes with Drp1 and sites of ER-mitochondria contact. This functional and spatial association of short Opa1 with mitochondrial division suggests that the processing of Opa1 can function to balance mitochondrial division and fusion dynamics.

### Mitochondrial motility and tethering: regulators of mitochondrial position

The activities of mitochondrial motility and tethering impact the overall cellular distribution of mitochondria. These activities are critical to ensure mitochondria are trafficked to and maintained at the cellular locations where they are needed.

#### Mitochondrial motility and tethering in yeast

In the simple polarized budding yeast cell, mitochondrial motility and tethering are critical to ensure that the daughter cell inherits and mother cell retains the essential mitochondrial compartment (Figure 4a). Mitochondria are actively transported to the growing bud via Myo2-driven transport along the actin cytoskeleton [76-82]. While transport is critical to place mitochondria in the bud, mother- and bud-specific mitochondrial tethers ensure that both cells retain part of the essential mitochondrial network [83-85]. Interestingly, daughters are born with a constant mitochondrial content to cell size ratio, suggesting that there is communication between mitochondrial transport and biogenesis and cell growth pathways in yeast [86].

**Figure 4:**
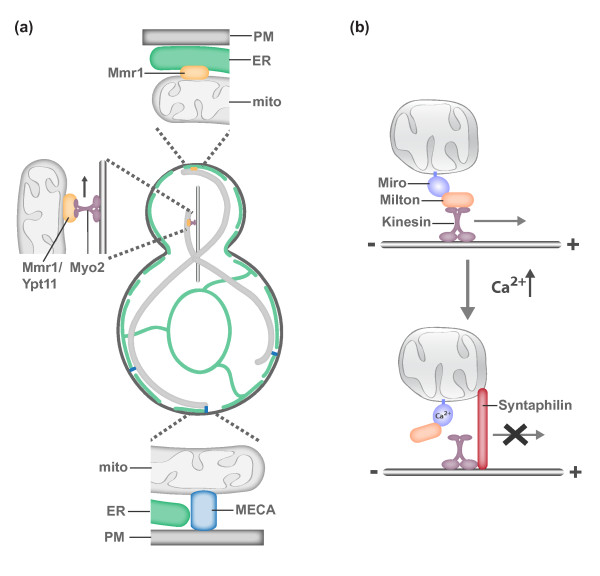
**Molecular models of mitochondrial motility and tethering. (a)** In yeast, mitochondria are actively transported to the growing bud via Myo2-driven transport along the actin cytoskeleton. Myo2-driven transport requires either Mmr1 or Ypt11 [76-82]. Mother- and bud-specific mitochondrial tethers ensure that both cells retain part of the essential mitochondrial compartment. The mother specific tether MECA (mitochondria-ER-cortex anchor) is composed of three membranes, the plasma membrane (PM), ER and mitochondria, and at least two proteins, Num1 and Mdm36 [84]. In addition to serving as a mitochondrial adaptor for Myo2, Mmr1 functions to tether mitochondria to ER sheets at the bud tip [83]. **(b)** A model for activity-dependent transport and tethering of mitochondria in axons. The conserved MOM Rho-like GTPase Miro and its binding partner Milton function as a mitochondrial receptor for kinesin. In active synaptic regions, Ca^2+^-binding by Miro triggers a confirmation change that disrupts kinesin-driven mitochondrial transport [98,99]. Ca^2+^-mediated confirmation changes have been proposed to disrupt the interaction between Miro/Milton with kinesin (shown here) or kinesin with microtubules. In response to neuronal activity (elevated Ca^2+^), syntaphilin is also recruited to mitochondria and functions as a static mitochondria-microtubule tether [100].

The yeast mitochondrial inheritance mechanism is also harnessed to contribute to mother-daughter age asymmetry, whereby daughter cells are born young despite the age of the mother [87]. In yeast, mitochondria retained in the mother cell have lower membrane and redox potential and higher superoxide levels than those inherited by the daughter [88]. How this asymmetry in mitochondrial function is achieved is not clear; however, selective transport and retention pathways likely play a role. Indeed, in the absence of the Myo2-mitochondrial adaptor/bud-specific tether Mmr1, mother-daughter age asymmetry is disrupted [88]. Whether the mother-specific tether also plays a role in the establishment of mother-daughter age asymmetry and how the selective transport and tethering of mitochondria are achieved are at this point unclear.

#### Mitochondrial motility and tethering in neurons

In more complex polarized cells such as neurons and immune cells, mitochondria must be actively transported to and maintained in active synaptic regions (Figure 4b), which have high demands for energy and Ca^2+^ buffering. Disrupting the synaptic translocation of mitochondria adversely affects both neuronal and immune cell function [6-8,89]. Due to the uniform, polarized arrangement of MTs in axons and the critical importance of mitochondrial positioning to neuronal function, neurons have proven to be an excellent model system to study regulated mitochondrial transport and tethering (for in depth review see [90,91]). In neurons, the kinesin Kif5 is the main motor driving anterograde mitochondrial transport, while the retrograde transport of mitochondria is driven by dynein. Adaptor proteins mediate the interactions between these cytoskeletal motors and mitochondria. The adaptor protein Milton/TRAK bridges the interaction between Kif5 and the MOM receptor Miro, a Rho-like GTPase with two GTPase domains, two Ca^2+^-binding EF hand motifs and two recently identified hidden EF-hand motifs that bind a helix that structurally mimics an EF hand ligand [92-94]. Miro likely also serves as the mitochondrial receptor for dynein as both anterograde and retrograde mitochondrial transport are disrupted in the absence of Miro and Miro’s binding partner TRAK interacts with both kinesin and dynein motors [95,96].

Miro has been proposed to function in activity-dependent regulation of mitochondrial transport via its ability to sense calcium [97-99]. A relatively small proportion of mitochondria are motile in neurons, and this motile population is further decreased in response to synaptic activity, which increases local Ca^2+^ concentrations. Ca^2+^ binding by Miro is proposed to trigger conformational changes that disrupt the interaction between Kif5 and MTs or Milton/TRAK and Kif5 and, consequently, disrupt mitochondrial transport [98,99]. Interestingly, retrograde transport does not take over in this circumstance, suggesting that dynein-dependent transport is also altered in response to elevated Ca^2+^ and/or a mitochondrial anchoring mechanism is activated. A recent study has elegantly dissected an activity-dependent anchoring mechanism [100]. The activity-dependent immobilization of axonal mitochondria requires the neuron-specific MOM protein syntaphilin [101]. In response to neuronal activity, syntaphilin is recruited to axonal mitochondria where it functions as an activity-regulated brake to mitochondrial transport. Syntaphilin competes with Milton/TRAK for Kif5 binding, and once bound to Kif5, syntaphilin inhibits the activity of the motor protein [100]. What mediates the activity-dependent recruitment of syntaphilin to axonal mitochondria and how the syntaphilin-mediated brake is released are outstanding questions. Interestingly, syntaphilin is not required for the activity-dependent immobilization of mitochondria in dendrites, indicating that a dendrite-specific tethering mechanism exists [100]. Consistently, mitochondrial motility in dendrites is primarily dynein-driven, supporting the need for a kinesin-independent tethering mechanism [96]. With the exception of the axonal-specific tether syntaphilin, the same motor and adaptor proteins and regulatory mechanisms that govern neuronal mitochondrial transport appear to be employed for mitochondrial transport in non-neuronal cell types [97,102].

#### The role of the ER in mitochondrial positioning

In yeast, the ER is a component of both the mother- and bud-specific mitochondrial tethers [83,84]. Whether the ER is a conserved component of positional mitochondrial tethers in other cell types remains to be determined. In activated T cells, both mitochondria and the ER are recruited to and maintained at the immune synapse [8,103]. Thus, ER-mitochondria tethers, such as Mfn2, may play a role in the synaptic translocation of mitochondria. Miro has also been localized to ER-mitochondria contacts in mammalian cells, raising the possibility that Miro-mediated ER-mitochondria tethering may also function in the positioning of mitochondria [50].

#### Additional functions of mitochondrial tethers

Mitochondrial tethers are not only important for positioning mitochondria relative to overall cellular structure but also play critical roles in positioning mitochondria relative to other organelles. The juxtaposition of membrane systems can facilitate the exchange of lipid, calcium and/or other small molecules between tethered compartments [104]. Indeed, the ER-mitochondria tether ERMES has been functionally linked to lipid transport between the ER and mitochondria in yeast [105], and ER-mitochondrial tethering by Mfn2 functions in Ca^2+^ signaling and lipid synthesis/transport between the two organelles [72,106]. In addition, Mfn2-mediated ER-mitochondrial contact plays a role in autophagosome biogenesis [107], and an ER-mitochondria tether composed of the ER protein Bap31 and mitochondrial anchored Fis1 is functionally associated with apoptosis as well as the removal of defective mitochondria by mitophagy [108,109]. Thus, mitochondrial tethers mediate functional interactions between the ER and mitochondria that are critical for many key cellular homeostatic pathways.

In addition to physical and functional connections to the ER, a recent study demonstrates that mitochondria are physically and functionally tethered to melanosomes, specialized lysosome-related organelles of pigment cells [110]. Mitochondria-melanosome tethering is mediated by Mfn2 and plays a role in melanosome biogenesis. Mitochondria also have close physical association with lipid droplets, although the molecular basis and functional consequences of this association are not known [111]. Given the number of ER-mitochondria contacts predicted for a single yeast cell alone is approximately 100 [112], it is likely that many additional inter-organelle tethers will be identified, many of which may be functionally distinct.

### Further integration of mitochondrial dynamics, motility and tethering pathways

Growing evidence suggests the activities of mitochondrial division, fusion, motility and tethering are interdependent and that the disruption of one activity can have indirect consequences on another. Indeed, attenuation of mitochondrial division disrupts the transport of mitochondria to neuronal and immune synapses, ultimately leading to detrimental effects on cellular function [6-8,89]. Mitochondrial tethering defects can reduce the rates of mitochondrial division [84,113,114], perhaps by disrupting the membrane tension required for DRP-mediated membrane scission [115]. In addition, mitochondrial transport is disrupted in the absence of the MOM fusion DRP Mfn2 [116]. Therefore, in addition to understanding each dynamic attribute in isolation, we must carefully consider the complex relationships that exist between the activities themselves and with the rest of the cell.
